# GC-MS Metabolic Profile and α-Glucosidase-, α-Amylase-, Lipase-, and Acetylcholinesterase-Inhibitory Activities of Eight Peach Varieties

**DOI:** 10.3390/molecules26144183

**Published:** 2021-07-09

**Authors:** Dasha Mihaylova, Ivelina Desseva, Aneta Popova, Ivayla Dincheva, Radka Vrancheva, Anna Lante, Albert Krastanov

**Affiliations:** 1 Department of Biotechnology, Technological Faculty, University of Food Technologies, 4002 Plovdiv, Bulgaria; a_krastanov@uft-plovdiv.bg; 2 Department of Analytical Chemistry and Physical Chemistry, Technological Faculty, University of Food Technologies, 4002 Plovdiv, Bulgaria; radka_vrancheva@yahoo.com; 3 Department of Catering and Tourism, Economics Faculty, University of Food Technologies, 4002 Plovdiv, Bulgaria; popova_aneta@yahoo.com; 4 AgroBioInstitute, Agricultural Academy, 8 Dr. Tsankov Blvd., 1164 Sofia, Bulgaria; ivadincheva@yahoo.com; 5 Department of Agronomy, Food, Natural Resources, Animals, and Environment—DAFNAE, Agripolis, University of Padova, 35020 Legnaro, Italy; anna.lante@unipd.it

**Keywords:** peach, amylase, glucosidase, lipase, acetylcholinesterase, enzyme inhibitory activities, GC-MS, principal component analysis, hierarchical cluster analysis

## Abstract

The inhibition of certain digestive enzymes by target food matrices represents a new approach in the treatment of socially significant diseases. Proving the ability of fruits to inhibit such enzymes can support the inclusion of specific varieties in the daily diets of patients with diabetes, obesity, Alzheimer’s disease, etc., providing them with much more than just valuable micro- and macromolecules. The current study aimed atidentifying and comparing the GC-MS metabolic profiles of eight peach varieties (“Filina”, “Ufo 4, “Gergana”, “Laskava”, “July Lady”, “Flat Queen”, “Evmolpiya”, and “Morsiani 90”) grown in Bulgaria (local and introduced) and to evaluate the inhibitory potential of their extracts towards α-glucosidase, α-amylase, lipase, and acetylcholinesterase. In order to confirm samples’ differences or similarities, principal component analysis (PCA) and hierarchical cluster analysis (HCA) were also applied to the identified metabolites. The results provide important insights into the metabolomic profiles of the eight peach varieties and represent a first attempt to characterize the peels of the peach varieties with respect to α-glucosidase-, α-amylase-, lipase-, and acetylcholinesterase-inhibitory activities. All of the studied peach extracts displayed inhibitory activity towards α-glucosidase (IC_50_: 125–757 mg/mL) and acetylcholinesterase (IC_50_: 60–739 mg/mL), but none of them affected α-amylase activity. Five of the eight varieties showed inhibitory activity towards porcine pancreatic lipase (IC_50_: 24–167 mg/mL). The obtained results validate the usefulness of peaches and nectarines as valuable sources of natural agents beneficial for human health, although further detailed investigation should be performed in order to thoroughly identify the enzyme inhibitors responsible for each activity.

## 1. Introduction

Digestive enzymes, primarily responsible for the breaking down of large molecules (carbohydrates, lipids, and proteins) into smaller, easily accessible ones, are a key component of the digestive system. In some cases, however, the inhibition of these enzymes forms part of the treatment of certain diseases [1]. Obesity and hyperglycemia (high blood glucose levels) are among the key metabolic changes that increase the risk of noncommunicable diseases (NCDs) [2]. Suppressing dietary sugar and fat absorption via α-glucosidase, α-amylase, and lipase inhibition is an important strategy against these diseases. It is not clear how α--amylase inhibitors influence hyperglycemia and obesity, despite studies showing that low serum amylase manifests in insufficient pancreatic insulin secretion and results in cardiometabolic conditions [3]. Lipase modulation can be seen as a promising treatment of obesity and type 2 diabetes, as lipase deficiency is associated with mitochondrial dysfunction and insulin resistance [4]. The α-glucosidase inhibitor miglitol, for instance, affects the regulation of bile acids involved in glucose and energy homeostasis [5]. NCDs affect people regardless of age, region, or country [2]. They are of serious concern, causing 41 million deaths annually, or more than 70% of total deaths worldwide, according to WHO [2].

Acetylcholinesterase (AChE) is another enzyme that is linked to socially important diseases. Its inhibition is an essential step to the treatment of several diseases, namely Alzheimer’s disease (AD), senile dementia, ataxia, myasthenia gravis, and Parkinson’s disease [6,7]. AChE inhibitors block the action of AChE and thus enhance the brain’s level of acetylcholine [8], which is otherwise hydrolyzed to acetic acid and choline. Several natural products with AChE-inhibitory activity have already been recognized as new multipotent AD drugs [9,10,11,12]. Compounds used for the treatment and prophylaxis of AD symptoms are mainly flavonoids and alkaloids. Galantamine, huperzine A, and curcumin are only a few of the natural compounds that have been documented and summarized as AChE inhibitors and memory-supporting elements [13]. Resveratrol, quercetin, and berberine are also considered to be protective contributors [14].

In the modern health context, the consumption of fruit and vegetables is a global priority [15]. Several reports have shown that adequate fruit and vegetable intake plays an important role in a healthy diet, while low fruit and vegetable intake is a risk factor for chronic diseases such as cancer, coronary heart disease (CHD), stroke, and cataract formation [16,17]. Furthermore, several fruits are likely to modulate metabolic risk factors like hypertension, dyslipidemia, diabetes, and obesity, and to inhibit the key pathological process of CHD and stroke—atherosclerosis [18,19,20,21,22,23]. Additionally, a diet rich in fruits and vegetables could prevent 20% of most types of cancers [16,24,25]. Several epidemiological and empirical studies have reported that the consumption of fruits and vegetables containing polyphenol compounds plays an important role in the inhibition of carbohydrate-hydrolyzing enzymes such as α-amylase, α-glucosidase [26,27,28,29], and acetylcholine esterase (AChE) [30].

Many factors (geographical conditions, growth phase, climatic influence, harvest time, etc.) influence the production of specific plant metabolites [31,32]. Thorough phytochemical profiling is important for the identification of the chemically diverse bioactive molecules and unknown compounds present in plants, and can provide valuable information about metabolic phenotypes [33,34]. The detection of these molecules can be performed with the use of various methods, e.g., gas chromatography coupled to mass spectrometry (GC-MS) [35]. Proper mathematical interpretation of the GC-MS results, like principal component analysis (PCA), allows the differentiation of plant species and can be applied for their chemotaxonomic characterization and classification [36,37].

**Figure 1:**
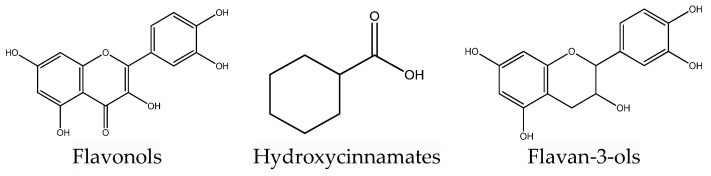
Some polyphenol compounds in peach fruit.

Peaches *(Prunus persica*) are not only an attractive stone fruit valued for direct consumption, but also a potential health-promoting food. Many metabolites contribute to this claim, but the primary effect is due to phytochemicals with mainly phenolic structures that exert various biological activities (Figure 1).

Polyphenols, carotenoids, and vitamin C are considered such contributors. Peaches have been reported to be rich in polyphenols although large variations are expected, usually based on factors like variety, climatic conditions, ripening stage, etc. [38]. Carotenoids, which also possess antioxidant activity, are important players in the intercellular communication and immune system activity [39,40]. In peaches, carotenoids are concentrated mostly in the peel but are also found in the flesh, especially of yellow fleshed-peaches [41,42].

Nowadays many studies focus on the beneficial effects of human health provoked by the consumption of foods with high polyphenolic content or target macro- and micro molecules. Peaches can be considered an interesting field of research as they exhibit several activities (antioxidant, protective, preventive, etc.), even though they may contain important substances in low quantities.

In this regard, the aim of the present study was to study and compare the GC-MS metabolic profiles of eight peach varieties grown in Bulgaria (local and introduced) as well as to evaluate the inhibitory potential of their extracts towards α-glucosidase, α-amylase, lipase, and acetylcholine esterase.

## 2. Results and Discussion

The peach is a delicious summer fruit that is extensively consumed worldwide. Its consumption is often associated with the intake of vitamins and minerals. Together with its organoleptic merits, this fruit possesses valuable components that promote beneficial body processes [43].

### 2.1. Gas Chromatography–Mass Spectrometry (GC-MS) Profiling of Polar and Nonpolar Compounds of Analyzed Peach Samples

Due to the specificity of the GC-MS analysis, prior segregation of the compounds can help to achieve a better metabolite profile. GC-MS is most useful in the identification of nonpolar compounds, as polar ones are thermolabile and have low volatility. Thus, segregationof the polar fraction aids in its further analysis [44]. The GC-MS-based analysis of a nonpolar primary separation followed by a polar secondary separation revealed the presence of 47 metabolites of different chemical classes (amino acids, carbohydrates, sugar alcohols, organic acids, and fatty and phenolic acids).

In polar fraction I, 18 amino acids were identified (Table 1), within which L-threonine (0.02–1.37 mg/g DW) and L-lysine (0.02–1.57 mg/g DW) were the dominant amino acids in all investigated peach varieties. The “Ufo 4” variety was the richest in total essential and nonessential amino acids (7.51 mg/g DW and 5.44 mg/g DW, respectively), and the “July Lady” had the lowest total amino acid content. Similar results were obtained in a previous analysis of amino acid content by HPLC-DAD [45]. In addition to L-threonine and L-lysine, eight other essential amino acids were determined (L-valine, L-leucine, L-isoleucine, L-methionine, L-phenylalanine, L-arginine, L-histidine, and L-tryptophan). L-leucine was not present in the “Filina”, “July Lady”, and “Evmolpiya” varieties, while L-histidine was absent in the “Flat Queen” and “July Lady”. The “Filina”, “Laskava”, and “Evmolpiya” varieties did not contain L-tryptophan.

Shikimic acid (1.76–2.62 mg/g DW) was the principal organic acid of the seven identified in the analyzed samples (Table 1). The highest total organic acid content was determined in the “Ufo 4” variety (9.33 mg/g DW). All peach samples also contained the valuable vitamin L-ascorbic acid in the range from 0.30 to 0.45 mg/g DW. In contrast to the current results, Famiani et al. [46] and Guo et al. [47] reported that the most abundant organic acids were malic, citric, and quinic acids. According to Colaric et al. [48], malic acid was the main organic acid (in the range from 3.82 to 8.05 g/kg fresh weight), and the content of shikimic acid was between 191 and 271 mg/kg fresh weight. These differences depend on the different peach varieties, and also on the tissue and stage of development, climatic and geographical conditions, and biotic and abiotic factors [33,34,46]. During the ripening of peaches, the amount of malate and citrate decreases as a result of the dissimilation and metabolism of stored organic acids [46]. Furthermore, Wu et al. [49] reported that shikimic acid concentration fluctuated differently with fruit development, and was generally lower in fruits with more leaves. Quinic and shikimic acids are known to be polyphenol precursors. In fruits, the amount of quinic acid is usually higher than that of shikimic acid [50], although this was not the case in the current results. The shikimic acid content points to a relationship with the phenolic acid content, which could suggest that this organic acid is linked to polyphenol synthesis. The shikimic acid pathway is also accountable for the biogenesis of aromatic amino acids, e.g., tryptophan, tyrosine, and phenylalanine [51].

Cell division, growth, respiration, storage, and reproduction are only some of the essential functions of a plant’s primary metabolites [52]. Amino acids are not only the constructive elements of proteins, but are also responsible for a fruit’s taste [53]. They can occur in a free form or in nonprotein compounds [54]. Amino acids have important nutritional value and are initial metabolites for the biosynthesis of various secondary metabolites, including polyphenols and aromatic and flavor compounds.

In accordance with a previous HPLC-DAD analysis of peach varieties [45,55], two monosaccharides (glucose and fructose) and one disaccharide (sucrose) were present in polar fraction I (Table 1). Sucrose, while prevalent in all samples, had the highest content in the “Laskava” variety. Glucose dominated over fructose in all investigated peach varieties. Glucose, fructose, sucrose, and sorbitol are also the major sugar components of other peach and nectarine varieties [47]. Sugars are responsible for a wide variety of metabolic pathways. They are known as energy producers and precursors for primary and secondary metabolites [56]. In addition to carbohydrates, the polar fraction was composed of two sugar alcohols (sorbitol and myo-inositol). Sorbitol was mostly present in the “Filina” variety (0.48 mg/g DW), and myo-inositol in “Flat Queen” and “Filina”. According to Robertson and Meredith [57], high-quality peaches have higher fructose and lower sorbitol and glucose concentrations compared to low-quality peaches.

Polar fraction II consisted of six phenolic acids (protocatechuic acid, trans-p-coumaric acid, trans-ferulic acid, trans-caffeic acid, trans-sinapic acid, and chlorogenic acid), with chlorogenic acid dominating (between 1.24 and 5.22 mg/g DW) in all peach samples (Table 2).

Phenolic acids are the most abundant plant secondary metabolites and have important physiological functions. They support plant growth and development, defense mechanisms, and pollinator attraction [58]. Polyphenols and amino acids are also substrates for the phenylpropanoid and lignin pathways during stone hardening [59]. Previous analysis of phenolic acids in the investigated peach varieties (HPLC-DAD) also revealed that chlorogenic acid was the major phenolic compound [45,55]. Similarly, other studies also reported that chlorogenic acid was one of the main phenolic acids in different peach varieties [41,60,61,62,63,64]. This phenolic acid possesses various valuable biological activities, such as anti-inflammatory [65], anticancer [66,67], antioxidant [68], antiepileptic, neuroprotective [69], antidiabetic [70], and antimicrobial activities [71], among others.

GC-MS-based analysis of the nonpolar fractions of the analyzed peach varieties revealed the presence of five saturated fatty acids (myristic acid, palmitic acid, arahidic acid, behenic acid, and stearic acid), and three unsaturated fatty acids (linoleic acid, oleic acid, and linolenic acid). The palmitic (between 2.86 and 7.94 mg/g DW) and linoleic acids (in the range of 1.77 and 4.92 mg/g DW) were the major ones (Table 3). Among the investigated peach samples, the “Laskava” variety accumulated the highest amount of both saturated and unsaturated fatty acids. Similarly, Duan et al. [72] reported that palmitic and linoleic acid were the predominant fatty acids in *Prunus persica* (L.) Batsch cv. “Shuangjiuhong” and cv. “Kawanakajima Hakuto”. The lipid compositions of fruit and vegetables have recently received great attention, especially for their essential fatty acids (linoleic, linolenic, and arachidic acids) [73]. These fatty acids play a natural preventive role in cardiovascular diseases and aid in the alleviation of other health problems, because they promote the reduction of both total and high-density lipoprotein (HDL) cholesterol [74]. Fruit species bear great similarities and differences in the fatty acid composition among varieties, even though fruit species in general have characteristic fatty acid compositions and profiles during development and ripening [72].

### 2.2. Principal Component Analysis (PCA) and Hierarchical Cluster Analysis (HCA) of GC-MS Data

**Figure 2:**
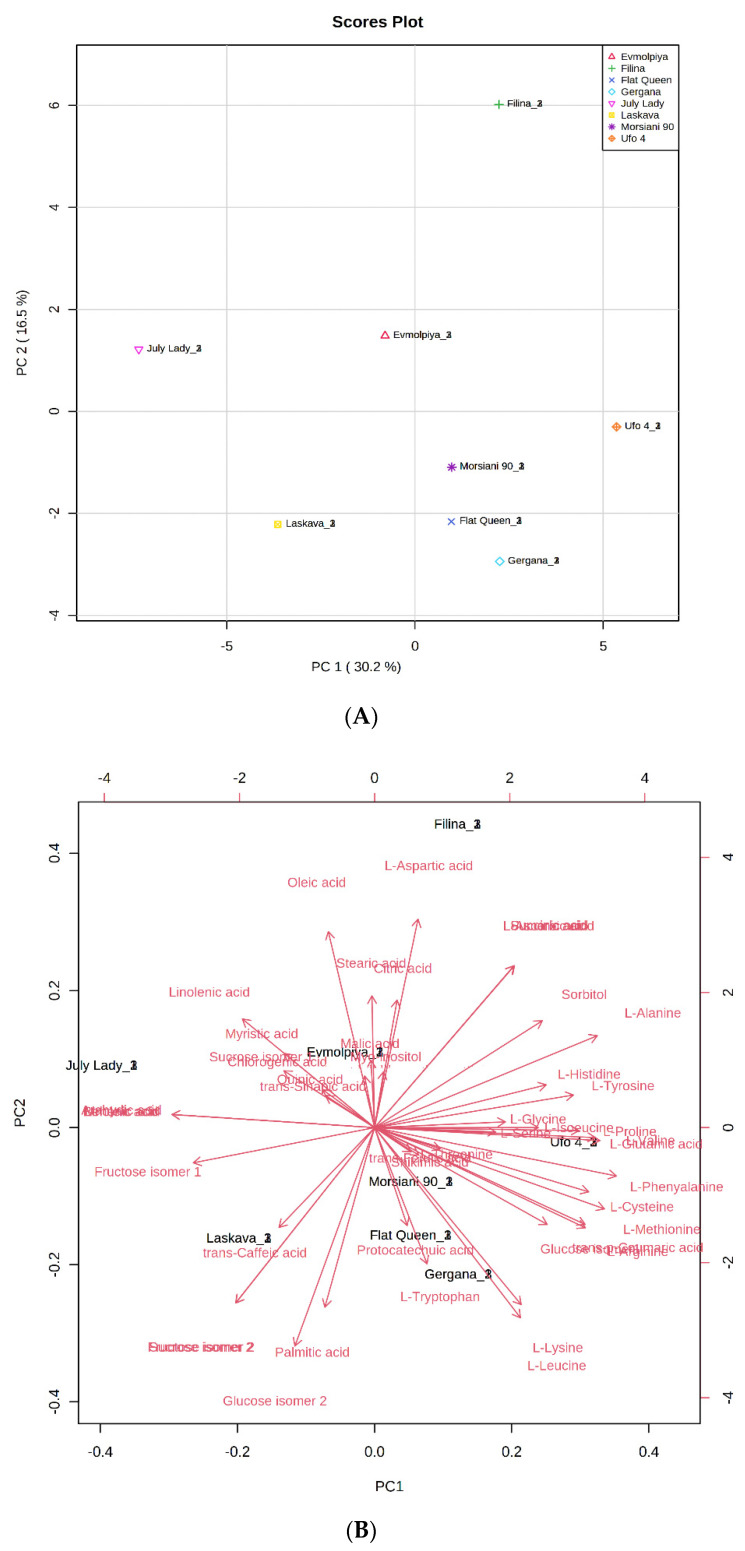
Principal component analysis (PCA) of GC-MS data of peach (*Prunus persica* L.) varieties’ metabolites. (**A**) Principal component score plot for the eight peach varieties. (**B**) Eigenvector load values of compounds identified in the eight peach varieties.

In order to confirm samples’ differences or similarities, principal component analysis (PCA) and hierarchical cluster analysis (HCA) were applied to the identified metabolites. According to the obtained PCA plot, the first two principal components, PC1 (30.2%) and PC2 (16.5%), accounted for 46.7% of the total variance of all identified compounds in the analyzed peach varieties (Figure 2). Metabolites with high positive scores in PC1, which distinguished “Filina” from the other varieties, were L-aspartic acid, succinic acid, and L-glutamic acid, sorbitol, L-alanine, L-tyrosine, L-histidine, L-glycine, L-proline, and myo-inositol. Oleic, stearic, citric, linolenic, myristic, malic, and trans-sinapic acids; sucrose isomer 1; and quinic, behenic, and arahydic acids showed high negative load scores in PC1 and distinguished the “Evmolpiya” and “July Lady” varieties from the other six. The “Laskava” variety was separated from the other samples by the high negative loading values of fructose isomer 1, trans-caffeic acid, sucrose isomer 2, fructose isomer 2, palmitic acid, and glucose isomer 2 in PC2. L-threonine, protocatechuic acid, shikimic acid, L-tryptophan, trans-p-coumaric acid, L-leucine, L-lysine, L-methionine, L-cysteine, L-phenylalanine, L-glutamic acid, L-valine, and L-isoleucine had high positive loadings values in PC2, and clearly differentiated the “Morsiani 90”, “Ufo 4”, “Flat Queen”, and “Gergana” varieties from the others. The PCA results displayed more variety-dependent levels of the identified metabolites than ripening stage dependence.

**Figure 3:**
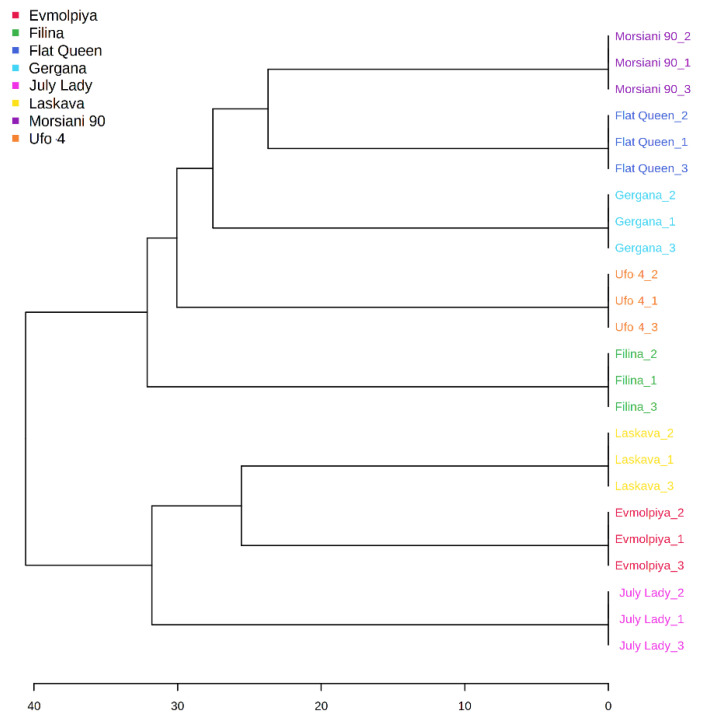
Clustering result of peach varieties, shown as a dendrogram (by Euclidean distance measure and Ward’s clustering algorithm).

**Figure 4:**
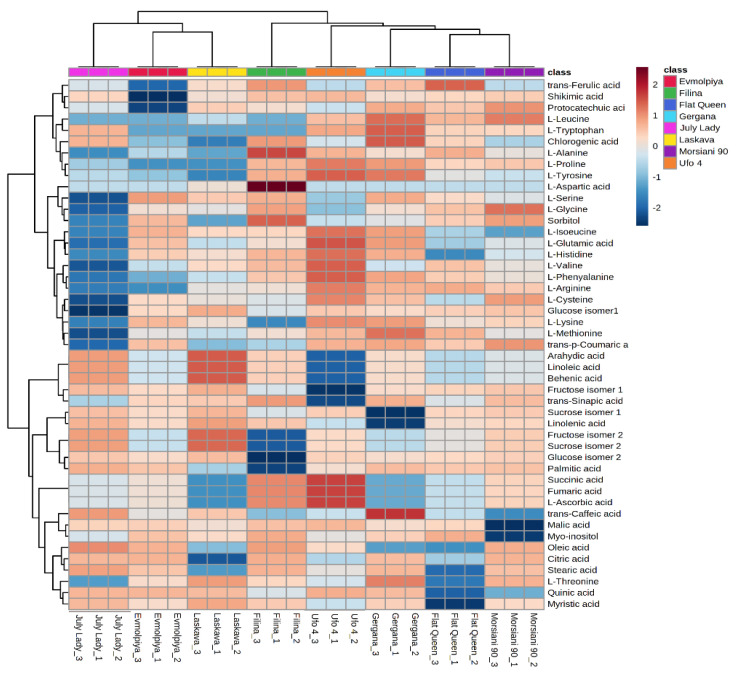
Clustering result of peach varieties shown as a heatmap. The color scale of the heatmap ranges from dark brown (value: +2) to dark blue (value: −2). The values were normalized by log_10_ transformation.

HCA was performed to outline the relationships between the analyzed varieties. According to the dendrogram and heatmap obtained, “Evmolpiya” had the highest phytochemical similarity to the “Laskava” variety, and these two varieties were grouped in the same cluster (Figure 3 and Figure 4). The observed cluster was associated with a higher amount of carbohydrates and a lower content of phenolic acids. The HCA also showed that “Morsiani 90” had the highest phytochemical similarity to “Flat Queen”. Their clustering together could be explained by the similar quantities of the identified metabolites. HCA also highlighted the highest metabolic diversity among the “July Lady” and “Morsiani 90” samples, with significant differences in the quantities of the identified metabolites. It should be underscored that “Flat Queen” and “Ufo 4” are flat peaches, and they were separated into different clusters because of the identified metabolite quantity differences, which could be explained by the different ripening stages—“Flat Queen” is late-ripening peach variety and “Ufo 4” is an early-ripening variety. The same results were obtained for “Gergana” and “Morsiani 90”. Both varieties are nectarines, but “Gergana” is an early-ripening variety while “Morsiani 90” is a late-ripening variety. This confirms that the ripening period has a direct link to the quantities of metabolites present in peaches.

PCA and HCA are statistical methods that are widely used to evaluate the clustering of different plant samples according to their chemical components [47,75,76,77].

### 2.3. Inhibitory Potential of Analyzed Peach Samples towards α-Glucosidase, α-Amylase, Lipase, and Acetylcholinesterase 

The presence in peaches of antioxidant compounds such as polyphenols is recognized by consumers. However, many diets exclude fruits because of their sugar content and their potential influence on blood glucose levels. At the same time, studies have demonstrated the relationship between polyphenol content and inhibitory activity towards some digestive enzymes, namely α-glucosidase, lipase, and α-amylase [29], as well as towards acetylcholinesterase [9,78,79]. The latter is the key enzyme in the treatment of Alzheimer’s-type dementia. Alpha-amylase is a secretory product of the salivary gland and pancreas. It hydrolyzes the initial breakdown of complex carbohydrates to oligo and disaccharides. The enzyme α-glucosidase catalyzes the final step of glucose absorption in the intestine during the process of carbohydrate digestion; thus, α-glucosidase inhibitors could slow the rapid utilization of dietary carbohydrates and suppress postprandial hyperglycemia [80].

The polyphenol contents of the eight peach varieties’ extracts were reported in prior studies [45,55]. Based on these results, the potential of the same extracts to inhibit the aforementioned enzymes was examined. The main hypothesis being tested was that the studied peach varieties can serve as a component in the therapeutic diet of people affected by diabetes, obesity, or Alzheimer’s, providing valuable information that will broaden the horizon for future research and further dissemination. This study is believed to be the first report on the inhibitory activity of Bulgarian peaches against these enzymes. In the current study, the whole fruits were subjected to extraction with two types of solvent, 80% aqueous methanol and water, while the peel was subjected only to extraction with 80% aqueous methanol.

The α-amylase- and α-glucosidase-inhibitory activities of different whole fruits varieties have been well studied. Although the same extracts have been shown to be more or less active towards both enzymes in most studies, exceptions have been reported. Quercetin has been shown to inhibit the activity of α-glucosidase but not α-amylase [81]. In contrast, the isoflavone daidzein inhibited porcine pancreatic α-amylase but had no effect on rat small-intestinal α-glucosidase [82]. In this study, no activity towards α-amylase was detected in any of the samples (Table 4), in contrast to other researchers’ findings concerning peach varieties grown in Poland [83]. The study of Kazeem et al. [84] claimed that the nature of α-amylase inhibition by phenolic components is competitive with the substrate, while the inhibition of α-glucosidase is noncompetitive, suggesting that the inhibitor binds to a separate site of the enzyme. It is most likely that the potential inhibitors in the current extracts formed weaker liaisons with α-amylase compared to the potato starch used as substrate. Therefore, no inhibition occurred. In the present study, the water extracts of “Filina”, “Gergana”, “Ufo 4”, “July Lady”, and “Laskava” were more effective than the methanolic ones towards α-glucosidase. All but one variety followed this trend. The methanolic extract of “Ufo 4” was the least effective (with an inhibitory concentration of 757 mg/mL), while the IC_50_ values of the other extracts varied from 201 to 498 mg/mL. Zhang et al. [85] reported tenfold better inhibitory activity of methanolic extracts, while Bento et al. [86] noted IC_50_ in the range from 11.7 to 35.8 µg/mL for Portuguese peach varieties. Nowicka et al. [83] reported comparably lower α-glucosidase-inhibitory concentrations (25.20–214.40 mg/mL). The differences between species of different varieties are mainly explained by genetic and environmental factors [87]. In addition, the extraction procedure can also target certain components and thus increase their concentrations in the final extract. It has been suggested that substances with a smaller sugar group attached inhibit digestive enzymes more successfully [88].

Lipase is the pancreatic enzyme that catalyzes the hydrolysis of triglycerides. “Morsiani 90” fruits did not contain any inhibitors of porcine pancreas lipase. The methanol extracts (ME) of “Gergana” and “July Lady” were the most potent, resulting in IC_50_ values of 31 and 39 mg/mL, respectively. In contrast, the water extracts (WE) of “Ufo 4” and “Laskava” were the least effective with 167 and 125 mg/mL. However, the same concentration of the extract exhibited lower activity towards lipase compared to the other tested enzymes. This substantiates the work of Nowicka et al. [83], who reported significantly lower results (0.07–2.06 mg/mL) compared to those established herein.

The inhibition of acetylcholinesterase is part of the treatment of Alzheimer’s disease. The serious side effects of synthetic inhibitors have forced the research community to search for natural alternatives. So far, galanthamine, huperzine A, and tacrine have been used for therapy [89]. Additionally, fruits and vegetables with proven target activities can be included in the daily menus of susceptible subjects. In this study, all extracts showed inhibitory activity towards AChE, with the most effective being the “Morsiani 90” ME (IC_50_–67 mg/mL) and WE (IC_50_–128 mg/mL) extracts. The ME of “Ufo 4” was the least active, with an IC_50_ of 739 mg/mL. The potential of *Prunus persica* L. water extracts to inhibit AChE effectively was also demonstrated in vivo [90]. Although it was thought that phenolic compounds are responsible for the inhibitory activity towards this enzyme [89], Rodríguez-Solana et al. [91] reported a negative correlation (according to Pearson correlation coefficients) in carob liqueurs. Nakagawa et al. [92] found no inhibitory activity of extracts of the peel and flesh of unripe fruits of “Akatsuki” and “Fastigiata” peach varieties. It could be suggested that the presence of caffeic and chlorogenic acids contribute to the AChE-inhibitory activity of the extracts, as both phenolic acids exhibit neuroprotective properties. The modulatory effect of caffeic and chlorogenic acids has been reported in several other studies [93,94]. It can be concluded that even low containing polyphenolic fractions exhibit an effect.

Within the available literature, the present study represents the first attempt to characterize the inhibitory activity of peach peel towards the studied enzymes. The peel is an underestimated part of the fruit. It is often removed prior to consumption or processing. However, it concentrates most of the fruit’s valuable compounds. The presence of phenolic compounds, with correlated antioxidant activity, has been reported to be two to three times higher in the peel than in the flesh [95,96]. In this study, none of the peel extracts affected α-amylase activity (Table 5), but all of them inhibited the activity of α-glucosidase. The highest inhibition respective to the lowest concentration was recorded for the “Gergana” variety extract: an IC_50_: of 125 mg/mL.

Five of the eight varieties displayed inhibitory activity towards the lipase from porcine pancreas. The lowest IC_50_: of 24 mg/mL was noted for the peel of the “Gergana” variety, while the highest was noted for “Morsiani 90” (IC_50_: 88 mg/mL). All five extracts displayed better effectiveness in the inhibition of lipase than of α-glucosidase or acetylcholinesterase.

In the current study, all peel extracts displayed inhibitory activity towards acetylcholinesterase. The lowest concentration that inhibited 50% of AChE was 60 mg/mL of the “Morsiani 90” peel extract. The Bulgarian variety “Evmolpiya” followed with an IC_50_ of 147 mg/mL. The white-fleshed “Flat Queen” peel extract seemed to be least effective (IC_50_: 487 mg/mL). The same extract also displayed the lowest total phenolic content, as reported previously [45].

## 3. Materials and Methods

### 3.1. Fruit Samples

The following eight peach and nectarine varieties, with early to late harvesting dates, were used: “Filina” (peach), “Ufo 4” (flat peach, white flesh), “Gergana” (nectarine), “Laskava” (peach), “July Lady” (peach), “Flat Queen” (flat peach, white flesh), “Evmolpiya” (peach), and “Morsiani 90” (nectarine). All were grown on the same plantation. “Filina”, “Gergana”, “Laskava”, and “Evmolpiya” are new Bulgarian varieties created at the Fruit Growing Institute, Plovdiv through interspecific hybridization. “Flat Queen”, “Morsiani 90”, “July Lady”, and “Ufo 4” are foreign varieties imported to Bulgaria.

No bactericides were applied to plants during testing.

The undamaged peach, nectarine, and flat fruit were harvested at eating ripeness in the Fruit Growing Institute, Plovdiv, BG (lat. 42.10384828045957 and long. 24.72164848814686). Fruits on the trees were considered ripe when the growth of the fruit had stopped and the fruit began to soften, exhibited a yellow or orange ground color (which is also representative for each variety), and was easily detached. Extraction procedures were performed and described for each analysis.

### 3.2. Gas Chromatographic–Mass Spectrometry Analysis (GC-MS) of Polar and Nonpolar Metabolites

#### 3.2.1. Extraction Procedure

A 50.0 mg measure of lyophilized and ground (electric mill (Tissue Lyser II, Qiagen, Hilden, Germany)) plant mater ial was mixed with 500.0 μL methanol (1.0 mg/mL), 50.0 μL ribitol (1.0 mg/mL), 3,5-dichloro-4-hydroxybenzoic acid (1.0 mg/mL), and nonadecanoic acid (1.0 mg/mL) as internal standards. The samples were vortexed for 10 s and incubated for 30 min at 70 °C. Subsequently, 500.0 μL chloroform and 300.0 μL distilled H_2_O were added after cooling to the ambient temperature. The samples were then vortexed for 10 s and centrifuged (10 min, 13,000 rpm). The polar and nonpolar fractions were prepared exactly as described by Vrancheva et al. [97]. The obtained fractions were subjected to GC-MS analysis.

#### 3.2.2. GC-MS Analysis

A 7890A gas chromatograph coupled with a 5975C (Agilent Technologies, Santa Clara, CA 95051, USA) (Agilent Technologiesinert XL EI/CI MSD at 70 eV (Agilent Technologies, Santa Clara, CA 95051, USA)) mass detector was used to perform the analysis. The conditions used for the separation of compounds followed the procedure described by Vrancheva et al. [77]. Results are presented as µg of respective internal standard equivalent per g dry weight (DW).

#### 3.2.3. Identification of the Metabolites

AMDIS software, version 2.64 (Automated Mass Spectral Deconvolution and Identification System, NIST, Gaithersburg, MD, USA) aided in the reading of the obtained mass spectra. For identification, the separated compounds were compared to their GC-MS spectra and Kovats retention index (RI) with reference compounds in the Golm Metabolome Database (http://csbdb.mpimp-golm.mpg.de/csbdb/gmd/gmd.html, accessed on 25 January 2021) and NIST’08 database (NIST Mass Spectral Database, PC-Version 5.0, 2008 from National Institute of Standards and Technology, Gaithersburg, MD, USA). The 2.64 AMDIS software recorded the RIs of the compounds with a standard n-hydrocarbon calibration mixture (C_8_–C_36_, Restek, Teknokroma, Spain).

### 3.3. Enzyme-Inhibitory Activities

#### 3.3.1. Extract Preparation

Methanol fruit extract (MEF): random samples of fresh whole fruits of each variety (approximately 20 g) were cut into small pieces and extracted with 80% aqueous methanol (methanol:water, 80:20, *v*/*v*, ratio 1:2.5) at 50 °C by ultrasonication for 30 min. The residues and the extracts were separated by filtration through filter paper; the obtained residues were re-extracted with a fresh portion of extractant (ratio 1:2) under the same conditions.

Water fruit extract (WEF): random samples of fresh whole fruits of each variety (approximately 20 g) were cut into small pieces and extracted with water (ratio 1:5) by ultrasonication at 50 °C for 15 min. The extract was then subjected to heat reflux extraction for 30 min, and afterwards the residues and the extracts were separated by filtration through filter paper.

Methanol peel extract (MEP): The peel of fresh fruits (approximately 15 g) was cut into small pieces and extracted with 80% aqueous methanol (methanol:water, 80:20, *v*/*v*, ratio 1:3.33) at 50 °C by ultrasonication for 30 min. The residues and the extracts were separated by filtration through filter paper; the obtained residues were re-extracted with a fresh portion of extraction (ratio 1:2) solvent under the same conditions.

The extracts recovered from each of the extraction procedure were further concentrated with a vacuum rotary evaporator (IKA RV10 digital, IKA HB 10 digital water bath IKA^®^-Werke GmbH & Co., Staufen im Breisgau, Germany) at 50 °C. The obtained semiliquid extracts were preserved at 4 °C until their use for further experiments, but with the storage not exceeding 7 days. Concentration was performed to more adequately assess biological activity. Sample concentration was performed between five and ten times, and this was taken into account when calculating the results. The volumes are in accordance with the wetting of the fruit parts during the extraction.

#### 3.3.2. α-Amylase (AM)-Inhibitory Assay

Each extract was mixed with enzyme solution (1:1, *v*/*v*) to obtain a final concentration of 1 U/mL α-amylase. The mixture was left for 15 min at 23 °C. The remaining α-amylase activity was performed exactly as descried by the Sigma Aldrich method [98]. The absorbance was measured at 540 nm. Enzyme without inhibitors was used as a negative control. The inhibition rate of α-amylase was assessed using the following formula (1):

α-amylase (%) = 100 − (A540 Blank corrected sample/A540 Blank corrected control) × 100(1)

The results are expressed as the concentration of extract (IC_50_) in mg/mL that inhibited 50% of α-amylase.

#### 3.3.3. α-Glucosidase (AG)-Inhibitory Assay

The reaction mixture contained 10 µL of extract (a minimum of five extract concentrations were tested in order to calculate the IC_50_) and 30 µL of α-glucosidase (0.1 U/mL, G5003-100UN, Sigma-Aldrich, Merck, Darmstadt, Germany). It was incubated for 15 min at 37 °C in a microplate reader (SPECTROstar Nano Microplate Reader, BMG LABTECH, Ortenberg, Germany). Afterwards, 25 µL of 1 mM 4-nitrophenyl-α-D-glucopyranoside (N 1377, Sigma-Aldrich, Merck, Darmstadt, Germany) was added. The reaction mixture was then shaken and incubated at 37 °C for 10 min. The reaction was terminated by adding 60 µL of 0.2 M sodium carbonate solution. Blanks were prepared by adding the extract after the termination of the reaction. The absorbance at 405 nm was measured using a microplate reader. Enzyme without inhibitor was used as a negative control. The α-glucosidase inhibition percentage of blank corrected data was assessed using the following formula (2):

% Inhibition = 100 − (A_405_ Blank corrected sample/A_405_ Blank corrected control) × 100 (2)

The results are expressed as concentration of extract (IC_50_) in mg/mL that inhibited 50% of α-glucosidase.

#### 3.3.4. Pancreatic-Lipase Inhibitory Assay

The in vitro pancreatic-lipase-inhibitory activity was determined as described by Saifuddin et al. [99] and Dobrev et al. [100] with slight modifications. The substrate was a mixture of 30 mg ρ-nitrophenyl palmitate (N2752, Sigma Aldrich, Merck, Darmstadt, Germany) solution in 10 mL isopropanol, 90 mL 0.05M Tris-HCl buffer with pH 7.2, 0.4 g Triton X-100, and 0.1 g gum arabic (G9752, Sigma Aldrich, Merck, Darmstadt, Germany). The reaction mixture consisted of 40 μL of the enzyme solution (15% lipase from porcine pancreas, L3126, Sigma Aldrich, Merck, Darmstadt, Germany in water) and 20 μL sample, which was incubated for 15 min at room temperature (25 °C). Subsequently, 20 μL of the mixture was withdrawn into a separate test tube containing 240 μL substrate and incubated at 35 °C for 2 h. The mixture was centrifuged at 4500× *g* for 10 min. The absorbance at 405 nm was measured. Enzyme without inhibitors was used as negative control. The inhibition percentage of pancreatic lipase was assessed using the following formula (3):

% Inhibition = 100 − (pNP_Sample_/pNP_Control_) × 100 (3)

where pNP is the amount of para-nitrophenol liberated after hydrolysis.

The pNP standard curve was generated in the range of 0.8–12.5 µg/mL.

The results are expressed as concentration of extract (IC_50_) in mg/mL that inhibited 50% of pancreatic lipase.

#### 3.3.5. Acetylcholineesterase (AChE)-Inhibitory Assay

The experimental conditions of the in vitro AChE-inhibitory assay were based on the method described by Lobbens et al. [101] with slight modifications. The acetylcholinesterase inhibitory assay was carried out in a 96-well microplate. Each well contained 30 µL AChE (final concentration of 0.05 U/mL, C3389-500U, Sigma Aldrich, Merck, Darmstadt, Germany), 125 µL 1.5 mM 5,5′-dithiobis(2-nitrobenzoic acid) (DTNB, D 218200, Sigma Aldrich, Merck, Darmstadt, Germany) dissolved in phosphate-buffered saline (PBS) pH 7.5, 45 µL PBS pH 7.5, and 25 µL test solution or 25 µL negative control (water). A blank sample was prepared by adding buffer instead of enzyme. The microplate was shaken for 10 s and left at 30 °C for 5 min. Subsequently, 30 µL of 7.5 mM acetylthiocholine (ATCI, 01480, Sigma Aldrich, Merck, Darmstadt, Germany) dissolved in water was added to each well and the absorbance was measured at 412 nm every 30 s for 1 min. The blank corrected data were plotted against time and the reaction rate (the slope of the plot) was calculated. Finally, the inhibition was calculated by comparing the reaction rate in the test solution compared to the negative control. The experiment was performed in triplicate. The inhibition was expressed as a percentage as follows (4):

%inhibition = 100 − (Slope_sample_/Slope_negative control_) × 100 (4)

The results are expressed as concentration of extract (IC_50_) in mg/mL that inhibited 50% of acetylcholinesterase.

### 3.4. Statistical Analysis

Analytical determinations were performed in triplicate and the results are expressed as means. Relevant statistical analyses of the data were performed using one-way ANOVA and a Tukey–Kramer post hoc test (α = 0.05), as described by Assaad et al. [102].

PCA and HCA of GC-MS data were conducted using MetaboAnalyst—a web-based platform (www.metaboanalyst.ca, accessed on 18 May 2021) [103]. The concentrations of the identified compounds were employed for PCA. All zero values were replaced with a small value (half of the minimum positive values in the original data) assumed to be the detection limit. First, PCA was applied in order to calculate the eigenvector load values and to identify the major statistically different components among the observations (samples). The GC-MS data were mean-centered and the PCA model was obtained at a confidence level of 95%. The GC-MS data were also subjected to HCA, which produced a dendrogram by Ward’s method of hierarchical clustering and Euclidean distance measurement between the analyzed samples. The values were normalized by log_10_ transformation.

## 4. Conclusions

This study provides important insights into the metabolomic profiles and inhibitory potential towards α-glucosidase, α-amylase, lipase, and acetylcholinesterase of eight peach varieties (“Filina”, “Ufo 4, “Gergana”, “Laskava”, “July Lady”, “Flat Queen”, “Evmolpiya”, and “Morsiani 90”).

GC-MS-based metabolite profiling and subsequent chemometric analyses helped to shed light on the different peach substances responsible for various important plant processes. The identified compounds varied from amino acids to phenolic compounds. The “Ufo 4” variety (flat peach, early ripening) dominated in its amino acid content (essential and nonessential), as well as total organic acid content. Predictably, sucrose was the most abundant carbohydrate found in all samples, with the highest values for the “Laskava” (peach, mid ripening) variety. The same variety also accumulated the highest amounts of both saturated and unsaturated fatty acids.

The results concerning the inhibitory potential of the peach varieties revealed a moderate inhibitory activity of the individual extracts. All of the studied peach extracts displayed inhibitory activity towards α-glucosidase and acetylcholinesterase. AChE-inhibitory activity may possibly be linked to the chlorogenic and caffeic acids present in the samples. None of the extracts affected the α-amylase activity. Five of the eight varieties showed inhibitory activity towards porcine pancreatic lipase. The resulting concentrations were much lower than those needed to inhibit α-glucosidase and acetylcholinesterase. For the first time in the literature, this works reports a GC-MS profile and attempt to characterize the inhibitory activities of the peel of peach varieties in respect to the aforementioned enzymes. The inhibitory effect requires further detailed investigation in order to reveal specific compounds and their contributions to the registered activity. As a future opportunity, in vivo studies would support the recorded observations herein.

## Figures and Tables

**Table 1:** Metabolites identified (mg/g DW) in polar fraction I of peach (*Prunus persica* L.) varieties.

№	RI	Name (LOD, ng/mL)	“Gergana”	“Flat Queen”	“Morsiani 90”	“Filina”	“July Lady”	“Laskava”	“Evmolpiya”	“Ufo 4”
**Essential amino acids**										
1	1208	L-Valine (10)	0.45 ± 0.01	0.18 ± 0.01	0.08 ± 0.01	0.21 ± 0.01	0.02 ± 0.00	0.12 ± 0.01	0.04 ± 0.00	0.81 ± 0.02
2	1266	L-Leucine (5)	0.08 ± 0.01	0.02 ± 0.00	0.05 ± 0.00	nd *	nd	0.01 ± 0.00	nd	0.12 ± 0.01
3	1285	L-Isoeucine (5)	0.39 ± 0.01	0.05 ± 0.00	0.21 ± 0.01	0.17 ± 0.01	0.01 ± 0.00	0.17 ± 0.01	0.30 ± 0.01	0.64 ± 0.01
4	1376	L-Threonine (15)	1.37 ± 0.02	0.70 ± 0.01	0.61 ± 0.01	0.30 ± 0.01	0.02 ± 0.00	0.88 ± 0.01	0.26 ± 0.01	1.14 ± 0.02
5	1515	L-Methionine (10)	0.59 ± 0.01	0.21 ± 0.01	0.05 ± 0.00	0.07 ± 0.01	0.01 ± 0.00	0.28 ± 0.01	0.05 ± 0.00	0.18 ± 0.01
6	1635	L-Phenyalanine (7)	0.42 ± 0.01	0.30 ± 0.01	0.22 ± 0.01	0.32 ± 0.01	0.05 ± 0.00	0.16 ± 0.01	0.09 ± 0.01	0.69 ± 0.01
7	1833	L-Arginine (10)	0.55 ± 0.01	0.61 ± 0.01	0.40 ± 0.01	0.25 ± 0.01	0.02 ± 0.00	0.21 ± 0.01	0.31 ± 0.01	1.04 ± 0.02
8	1910	L-Lysine (8)	1.26 ± 0.02	0.36 ± 0.01	0.52 ± 0.01	0.27 ± 0.01	0.02 ± 0.00	0.36 ± 0.01	0.83 ± 0.01	1.57 ± 0.02
9	2144	L-Histidine (10)	0.62 ± 0.01	nd	0,18 ± 0.01	0.56 ± 0.01	nd	0.30 ± 0.01	0.42 ± 0.01	1.21 ± 0.02
10	2211	L-Tryptophan (15)	0.22 ± 0.01	0.07 ± 0.01	0.07 ± 0.01	nd	0.10 ± 0.01	nd	nd	0.11 ± 0.01
Total essential amino acids, mg/g DW			5.95	2.50	2.39	2.15	0.25	2.49	2.3	7.51
**Nonessential amino acids**										
11	1097	L-Alanine (5)	0.15 ± 0.01	0.34 ± 0.01	0.13 ± 0.01	1.03 ± 0.02	0.02 ± 0.00	0.25 ± 0.01	0.06 ± 0.00	0.31 ± 0.01
12	1293	L-Proline (15)	0.44 ± 0.01	0.1 ± 0.00	0.17 ± 0.01	0.24 ± 0.01	0.01 ± 0.00	nd	nd	0.77 ± 0.01
13	1299	L-Glycine (5)	0.12 ± 0.01	0.20 ± 0.01	0.57 ± 0.02	0.33 ± 0.01	nd	0.09 ± 0.01	0.11 ± 0.01	0.26 ± 0.01
14	1351	L-Serine (10)	0.91 ± 0.01	0.53 ± 0.01	0.3 ± 0.01	0.87 ± 0.01	0.04 ± 0.00	0.64 ± 0.01	1.04 ± 0.01	1.82 ± 0.02
15	1508	L-Aspartic acid (5)	nd	nd	nd	1.68 ± 0.03	nd	0.10 ± 0.01	nd	nd
16	1550	L-Cysteine (15)	0.02 ± 0.00	0.01 ± 0.00	0.03 ± 0.00	0.01 ± 0.00	nd	0.01 ± 0.00	0.02 ± 0.00	0.04 ± 0.00
17	1609	L-Glutamic acid (6)	0.77 ± 0.01	0.20 ± 0.00	0.32 ± 0.01	0.41 ± 0.01	0.08 ± 0.01	0.20 ± 0.00	0.57 ± 0.01	1.26 ± 0.02
18	1930	L-Tyrosine (9)	0.64 ± 0.01	0.04 ± 0.00	0.02 ± 0.00	0.23 ± 0.01	0.02 ± 0.00	nd	0.08 ± 0.01	0.98 ± 0.01
Total nonessential amino acids, mg/g DW			3.05	1.42	1.54	4.80	0.17	1.29	1.88	5.44
**Organic acids**										
19	1305	Succinic acid (7)	0.80 ± 0.01	0.88 ± 0.01	0.97 ± 0.01	1.07 ± 0.02	0.91 ± 0.02	0.77 ± 0.01	0.94 ± 0.01	1.15 ± 0.01
20	1344	Fumaric acid (7)	0.60 ± 0.01	0.66 ± 0.01	0.73 ± 0.02	0.80 ± 0.01	0.68 ± 0.01	0.58 ± 0.01	0.71 ± 0.01	0.86 ± 0.01
21	1477	Malic acid (9)	1.12 ± 0.02	1.23 ± 0.02	1.35 ± 0.02	1.49 ± 0.02	1.26 ± 0.02	1.07 ± 0.01	1.31 ± 0.01	1.60 ± 0.02
22	1818	Shikimic acid (10)	1.83 ± 0.02	2.02 ± 0.02	2.22 ± 0.02	2.44 ± 0.04	2.07 ± 0.02	1.76 ± 0.02	2.15 ± 0.03	2.62 ± 0.02
23	1841	Citric acid (12)	0.94 ± 0.01	1.03 ± 0.01	1.13 ± 0.02	1.25 ± 0.03	1.06 ± 0.01	0.90 ± 0.01	1.10 ± 0.01	1.34 ± 0.02
24	1855	Quinic acid (5)	0.91 ± 0.01	1.00 ± 0.01	1.10 ± 0.02	1.21 ± 0.02	1.03 ± 0.01	0.87 ± 0.01	1.07 ± 0.01	1.30 ± 0.02
25	1946	L-Ascorbic acid (20)	0.32 ± 0.01	0.35 ± 0.01	0.38 ± 0.01	0.42 ± 0.01	0.36 ± 0.01	0.30 ± 0.01	0.37 ± 0.01	0.45 ± 0.01
Total organic acids, mg/g DW			6.52	7.17	7.89	8.67	7.37	6.27	7.65	9.33
**Sugar alcohols**										
26	1932	Sorbitol (10)	0.36 ± 0.01	0.39 ± 0.01	0.43 ± 0.01	0.48 ± 0.01	0.27 ± 0.01	0.29 ± 0.01	0.41 ± 0.01	0.34 ± 0.01
27	2034	Myo-inositol (10)	0.21 ± 0.01	0.27 ± 0.01	0.25 ± 0.01	0.28 ± 0.01	0.13 ± 0.01	0.19 ± 0.01	0.23 ± 0.01	0.15 ± 0.01
**Carbohydrates**										
28	1856	Fructose isomer 1 (15)	1.99 ± 0.02	2.19 ± 0.02	2.41 ± 0.02	1.43 ± 0.02	2.65 ± 0.02	2.91 ± 0.02	2.04 ± 0.02	2.33 ± 0.03
29	1865	Fructose isomer 2 (10)	0.69 ± 0.01	0.76 ± 0.01	0.83 ± 0.02	0.49 ± 0.01	0.92 ± 0.01	1.01 ± 0.01	0.71 ± 0.01	0.81 ± 0.01
30	1881	Glucose isomer 1 (10)	3.79 ± 0.04	4.17 ± 0.04	4.59 ± 0.03	2.72 ± 0.04	5.05 ± 0.05	5.56 ± 0.04	3.89 ± 0.05	4.44 ± 0.04
31	1901	Glucose isomer 2 (10)	2.86 ± 0.02	3.14 ± 0.03	3.46 ± 0.03	2.05 ± 0.03	3.80 ± 0.02	4.18 ± 0.04	2.93 ± 0.04	3.35 ± 0.03
32	2620	Sucrose isomer 1 (alpha-D-Glc-(1.2)-beta-D-Fru) (15)	6.12 ± 0.04	6.73 ± 0.04	7.41 ± 0.05	4.39 ± 0.04	8.15 ± 0.07	8.96 ± 0.08	6.27 ± 0.07	7.17 ± 0.06
33	2833	Sucrose isomer 2(alpha-D-Glc-(1.2)-beta-D-Fru) (15)	3.55 ± 0.03	3.91 ± 0.03	4.30 ± 0.04	2.55 ± 0.02	4.73 ± 0.03	5.20 ± 0.04	3.64 ± 0.04	4.16 ± 0.04
Total carbohydrates, mg/g DW			19.00	20.90	23.00	13.63	25.30	27.82	19.48	22.26

* nd—not detected.

**Table 2:** Metabolites identified (mg/g DW) in polar fraction II of the peach (*Prunus persica* L.) varieties.

№	RI	Name (LOD, ng/mL)	“Gergana”	“Flat Queen”	“Morsiani 90”	“Filina”	“July Lady”	“Laskava”	“Evmolpiya”	“Ufo 4”
1	1836	Protocatechuic acid (10)	0.27 ± 0.01	0.18 ± 0.01	0.40 ± 0.01	0.11 ± 0.01	0.06 ± 0.01	0.16 ± 0.01	0.20 ± 0.01	0.05 ± 0.00
2	1945	trans-p-Coumaric acid (20)	0.21 ± 0.01	0.12 ± 0.01	0.27 ± 0.01	0.23 ± 0.01	0.19 ± 0.01	0.14 ± 0.01	0.14 ± 0.01	0.18 ± 0.01
3	2103	trans-Ferulic acid (5)	0.13 ± 0.01	0.20 ± 0.01	0.08 ± 0.01	0.16 ± 0.01	0.09 ± 0.01	0.11 ± 0.01	0.04 ± 0.00	0.08 ± 0.01
4	2140	trans-Caffeic acid (20)	0.19 ± 0.01	0.07 ± 0.01	0.04 ± 0.01	0.05 ± 0.01	0.12 ± 0.01	0.10 ± 0.01	0.08 ± 0.01	0.07 ± 0.01
5	2254	trans-Sinapic acid (10)	0.09 ± 0.01	0.05 ± 0.01	0.08 ± 0.01	0.11 ± 0.01	0.03 ± 0.01	0.07 ± 0.01	0.07 ± 0.01	0.09 ± 0.01
6	3191	Chlorogenic acid (15)	5.22 ± 0.03	3.16 ± 0.03	2.00 ± 0.02	4.13 ± 0.03	3.67 ± 0.03	1.24 ± 0.01	1.78 ± 0.02	2.40 ± 0.02
Total phenolic acids, mg/g DW			6.11	3.78	2.87	4.79	4.16	1.82	2.31	2.87 ± 0.02

**Table 3:** Metabolites identified (mg/g DW) in the nonpolar fraction of peach (*Prunus persica* L.) varieties.

№	RI	Name (LOD, ng/mL)	“Gergana”	“Flat Queen”	“Morsiani 90”	“Filina”	“July Lady”	“Laskava”	“Evmolpiya”	“Ufo 4”
**Saturated fatty acids**										
1	1719	Tetradecanoic acid (Myristic acid) (8)	0.47 ± 0.02	0.38 ± 0.03	0.42 ± 0.02	0.49 ± 0.02	0.58 ± 0.02	0.68 ± 0.02	0.41 ± 0.02	0.24 ± 0.01
2	1926	n-Hexadecanoic acid (Palmitic acid) (15)	5.51 ± 0.04	4.46 ± 0.03	4.96 ± 0.04	5.80 ± 0.04	6.79 ± 0.06	7.94 ± 0.05	4.76 ± 0.04	2.86 ± 0.02
3	2311	n-Eicosanoic acid (Arahydic acid) (15)	1.05 ± 0.01	0.85 ± 0.03	0.95 ± 0.02	1.11 ± 0.01	1.30 ± 0.02	1.52 ± 0.02	0.91 ± 0.02	0.55 ± 0.02
4	2408	n-Docosanoic acid (Behenic acid) (5)	1.31 ± 0.02	1.06 ± 0.02	1.18 ± 0.02	1.38 ± 0.02	1.61 ± 0.01	1.89 ± 0.02	1.13 ± 0.01	0.68 ± 0.02
5	2247	n-Octadecanoic acid (Stearic acid) (20)	2.30 ± 0.02	1.86 ± 0.02	2.07 ± 0.02	2.42 ± 0.02	2.83 ± 0.03	3.31 ± 0.02	1.99 ± 0.02	1.19 ± 0.01
Total saturated fatty acids, mg/g DW			10.64	8.61	9.58	11.20	13.1	15.34	9.20	
**Unsaturated fatty acids**										
6	2095	9,12-(Z,E)-Octadecadienoic acid (Linoleic acid) (6)	3.42 ± 0.02	2.77 ± 0.02	3.07 ± 0.04	3.60 ± 0.03	4.21 ± 0.03	4.92 ± 0.02	2.95 ± 0.03	1.77 ± 0.02
7	2099	9-(Z)-Octadecenoic acid (Oleic acid) (5)	1.79 ± 0.02	1.45 ± 0.02	1.61 ± 0.01	1.89 ± 0.01	2.21 ± 0.03	2.58 ± 0.02	1.55 ± 0.02	0.93 ± 0.02
8	2103	9,12,15-(Z,Z,Z)-Octadecatrienoic acid (Linolenic acid) (10)	0.62 ± 0.01	0.50 ± 0.01	0.56 ± 0.01	0.65 ± 0.01	0.76 ± 0.01	0.89 ± 0.01	0.54 ± 0.01	0.32 ± 0.01
Total unsaturated fatty acids mg/g DW			5.83	4.72	5.24	6.14	7.18	8.39	5.04	3.02

**Table 4:** Enzyme-inhibitory activities (α-glucosidase, lipase, α-amylase, and acetylcholinesterase (AChE)) of eight peach varieties’ fruit extracts (methanol (ME) and water (WE)), IC_50_, mg/mL.

Variety	Extract	α-Glucosidase	Lipase	α-Amylase	AChE
“Gergana”	ME	299 ^g^	31	-	129 ^i^
	WE	242 ^i^	-	-	304 ^e^
“Flat Queen”	ME	434 ^c^	68 ^e^	-	205 ^g^
	WE	487 ^b^	-	-	217 ^g^
“Morsiani 90”	ME	310 ^f^	-	-	67 ^j^
	WE	399 ^d^	-	-	128 ^i^
“Filina”	ME	366 ^e^	-	-	254 ^f^
	WE	273 ^h^	92 ^c^	-	253 ^f^
“July Lady”	ME	302 ^g^	39 ^f^	-	195 ^g^
	WE	276 ^h^	80 ^d^	-	321 ^d^
“Laskava”	ME	498 ^b^	-	-	199 ^g^
	WE	324 ^f^	125 ^b^	-	520 ^b^
“Evmolpiya”	ME	351 ^e^	100 ^c^	-	166 ^h^
	WE	402 ^d^	88 ^c^	-	176 ^h^
“Ufo 4”	ME	757 ^a^	-	-	739 ^a^
	WE	201 ^j^	167 ^a^	-	334 ^c^

“-” not detected; values are means, *n* = 3 per treatment group. Different letters in the same column indicate statistically significant differences (*p* < 0.05), according to ANOVA (one-way) and the Tukey test.

**Table 5:** Enzyme-inhibitory activities (α-glucosidase, lipase, α-amylase, and acetylcholinesterase (AChE)) of eight peach varieties’ peel extracts, IC_50_, mg/mL.

Variety	α-Glucosidase	Lipase	α-Amylase	AChE
“Gergana”	125 ^f^	24 ^d^	-	238 ^c^
“Flat Queen”	272 ^c^	-	-	487 ^a^
“Morsiani 90”	351 ^b^	88 ^a^	-	60 ^h^
“Filina”	275 ^c^	-	-	221 ^d^
“July Lady”	157 ^e^	65 ^c^	-	263 ^b^
“Laskava”	282 ^c^	81 ^b^	-	162 ^f^
“Evmolpiya”	243 ^d^	61 ^c^	-	147 ^g^
“Ufo 4”	739 ^a^	-	-	197 ^e^

“-” not detected; values are means, *n* = 3 per treatment group. Different letters in the same column indicate statistically significant differences (*p* < 0.05), according to ANOVA (one-way) and the Tukey test.

## Data Availability

The data presented in this study are available on request from the corresponding author.

## Sample Availability

Samples of the compounds are available from the authors.
